# Mechanically tightening, untying and retying a protein trefoil knot by single-molecule force spectroscopy†

**DOI:** 10.1039/d0sc02796k

**Published:** 2020-10-19

**Authors:** Han Wang, Hongbin Li

**Affiliations:** Department of Chemistry, University of British Columbia Vancouver BC V6T 1Z1 Canada hongbin@chem.ubc.ca

## Abstract

Knotted conformation is one of the most surprising topological features found in proteins, and understanding the folding mechanism of such knotted proteins remains a challenge. Here, we used optical tweezers (OT) to investigate the mechanical unfolding and folding behavior of a knotted protein *Escherichia coli* tRNA (guanosine-1) methyltransferase (TrmD). We found that when stretched from its N- and C-termini, TrmD can be mechanically unfolded and stretched into a tightened trefoil knot, which is composed of *ca.* 17 residues. Stretching of the unfolded TrmD involved a compaction process of the trefoil knot at low forces. The unfolding pathways of the TrmD were bifurcated, involving two-state and three-state pathways. Upon relaxation, the tightened trefoil knot loosened up first, leading to the expansion of the knot, and the unfolded TrmD can then fold back to its native state efficiently. By using an engineered truncation TrmD variant, we stretched TrmD along a pulling direction to allow us to mechanically unfold TrmD and untie the trefoil knot. We found that the folding of TrmD from its unfolded polypeptide without the knot is significantly slower. The knotting is the rate-limiting step of the folding of TrmD. Our results highlighted the critical importance of the knot conformation for the folding and stability of TrmD, offering a new perspective to understand the role of the trefoil knot in the biological function of TrmD.

## Abstract 2

Optical tweezers are used to stretch a knotted protein along different directions to probe its unfolding–folding behaviors, and the conformational change of its knot structure.

## Graphical abstract

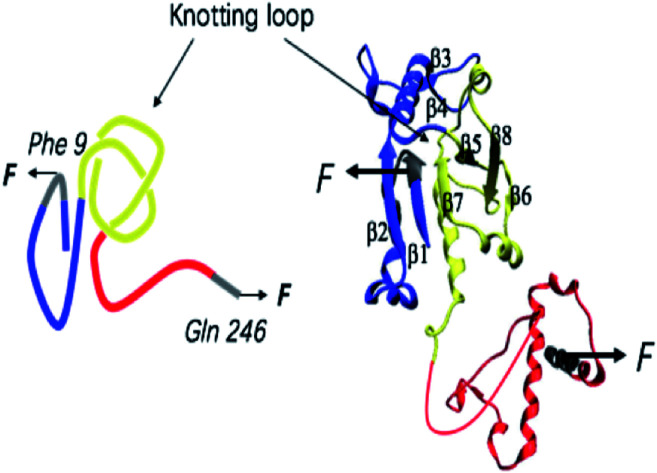

## Introduction

A protein knot is one of the most remarkable features found in proteins over the last two decades, and it adds a layer of topological complexity to the protein folding problem.^1–7^ More than 1300 proteins with knotted or slipknotted conformations have been identified from proteins whose three-dimensional structures are deposited in Protein Data Bank (PDB), including trefoil (3_1_), figure-of-eight (4_1_), Gordian (5_2_) and stevedore (6_1_) knots.^5,8,9^ These knots in proteins are believed to be functionally relevant, as well as provide extra structural stability to proteins.^9–12^ Understanding the molecular mechanism *via* which knotted proteins overcome the topological barriers to fold represents a significant challenge.^13^

Both experimental,^8,14–18^ including ensemble and single-molecule measurements, and computational studies^19–23^ have started to provide invaluable mechanistic insights into the folding of knotted/slipknotted proteins. It has been shown that such knotted proteins are able to overcome the high topological barrier to knot themselves and fold, although generally very slowly.^8,15,18,24,25^ Molecular dynamics (MD) simulations are of great importance to understanding the molecular mechanism of the folding of these proteins. These studies have revealed the complexity of the folding of knotted proteins and proposed several possible knotting mechanisms, including threading, slipknotting, mouse trapping and folding on ribosomes, to offer important insights into the folding mechanism of such knotted proteins at the molecular level.^20–23^

*Escherichia coli* tRNA (guanosine-1) methyltransferase D (TrmD) (PDB: 1P9P) possesses a deep trefoil knot within its protein structure.^26–31^ TrmD is responsible for the methylation of G37 in tRNA containing the sequence of G36pG37, which is critical to preventing frameshifting in the protein translation process.^26^ TrmD catalyzes the modification process by using *S*-adenosyl-l-methionine (AdoMet) as the methyl donor, and it is suggested that the conserved knot structure is key to accommodating the adenosine moiety of AdoMet by the loosening and retightening of the trefoil knot during the methylation process.^27^ Therefore, understanding the folding mechanism of TrmD as well as the structural dynamics of its knotted structure is not only important for elucidating the general folding mechanism underlying knotted proteins, but also key to understanding the functional role of the trefoil knot in the biological function of TrmD.

**Fig. 1:**
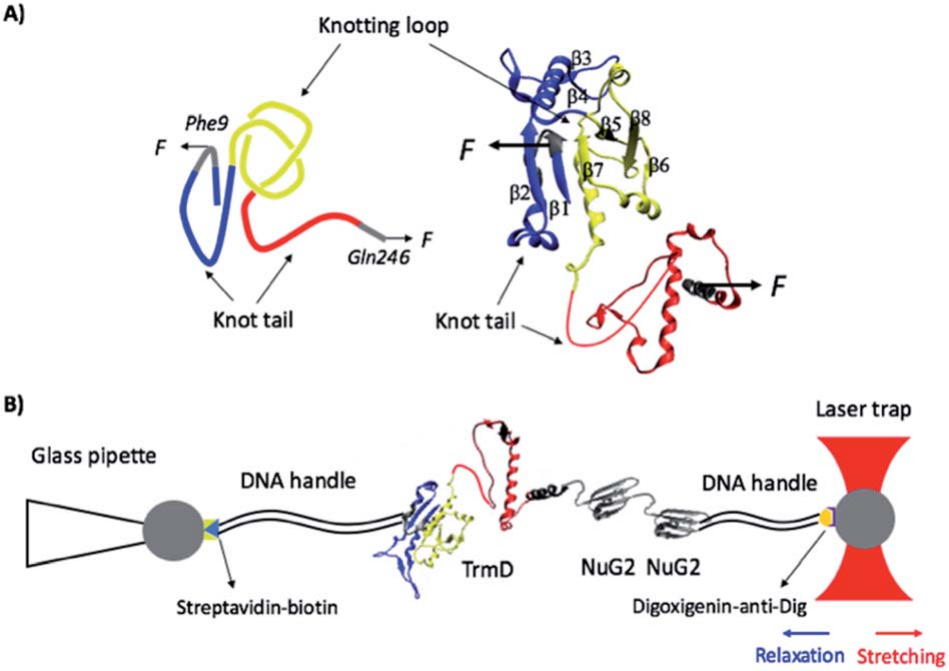
(A) The three-dimensional structure of the knotted protein TrmD (PDB code: 1P9P). TrmD is composed of two subdomains: the N-terminal Rossman fold with the motif of alternating β strand–α helix–β strand (residues 1–161, colored in blue and yellow) and the C-terminal α helical domain (residues 162–246, colored in red). The knotting loop is colored in yellow (residues 81–161), and the knot tails are colored in blue and red. Residues 9 and 246, along which TrmD is stretched in the OT experiment, are colored in grey. (B) Schematics of the experimental setup of optical tweezers. Protein chimera TrmD_9,246_–(NuG2)_2_ is coupled with two DNA handles *via* thio-maleimide chemistry. The NuG2 domain serves as a fingerprint for identification of single-molecule stretching events. This DNA–protein chimera is then held by two different polystyrene beads, with streptavidin and anti-digoxigenin modified on their surface, respectively. One bead is held by a glass pipette and the other is trapped by the OT. Stretching or relaxation of the protein chimera can be achieved by moving the laser trap.

TrmD is an α/β protein and its three dimensional structure can be readily divided into two subdomains (Fig. 1):^26^ the N-terminal α/β assembly domain (residues 1–161) and the C-terminal α helical domain (residues 162–246, colored in red). The N-terminal α/β assembly domain is a fold related to the Rossman fold, with a rather compact globular structure, and the C-terminal α helix domain consists of a long disordered polypeptide coil and four relatively flexible α helixes. TrmD contains a deep trefoil knot in its structure, with residues 81–161 (colored in yellow) serving as the knot core (also called the knotting loop), and residues 1–80 (colored in blue) and 162–246 (colored in red) as knot tails. Thus, TrmD serves as an ideal model system for investigating the folding–unfolding mechanism of trefoil-knotted proteins.

Amongst the experimental techniques used to study knotted and slipknotted proteins, single-molecule force spectroscopy (SMFS) is of particular advantages due to its ability to manipulate the knot/slipknot structure by directly stretching the structure, offering a unique perspective to study the knotting/unknotting process of these proteins.^8,16,32–35^ Atomic force microscopy (AFM) and optical tweezers (OT)-based SMFS techniques have been exploited for such studies. Due to the relatively low force resolutions (∼10 pN), AFM was mainly used to probe the unfolding/untying mechanism of knotted and slipknotted proteins, and direct observation of their folding was largely not possible in these studies.^16,32,35^ Due to its superb resolution in force (0.1 pN) and long-term stability,^8,36–40^ OT has just been utilized to investigate the folding/unfolding mechanism of the 5_2_-knotted protein ubiquitin C-terminal hydrolase isoenzyme L1 (UCH-L1) and slipknotted proteins AFV3-109 and pyruvoyl-dependent arginine decarboxylase (PADC).^8,33,34^ Given the diversity of knots/slipknots in proteins, much more can be learned about their folding mechanisms using the OT-based SMFS technique. Due to the simplicity of the trefoil knot, TrmD serves as an ideal model system for the OT study.

Here, we combined OT with protein engineering techniques to investigate the unfolding and folding mechanisms of TrmD. Our results showed that upon stretching from its N- and C-termini, TrmD can be mechanically unfolded and stretched into an unfolded polypeptide chain with a tightened trefoil knot. Both two-state and three-state mechanical unfolding pathways were observed. Upon relaxation, the tightened knot can be loosened by thermal energy and the unfolded polypeptide can fold back to its native state efficiently. The folding of TrmD from such an unfolded state containing a knot is fast and robust. Distinct from a typical two-state folding process, the folding of TrmD from an unstructured polypeptide containing a knot involves a loosening process of the tightened trefoil knot prior to the folding into its native state. In addition, by using a truncation mutant of TrmD, we unfolded TrmD and untied the trefoil knot. Our results showed that the folding of TrmD from such an unknotted and unfolded state was much slower, with the knotting process as the rate-limiting step during the folding of the whole knotted protein.

## Materials and methods

### Protein engineering

The wild-type TrmD contains two cysteine residues in its sequence (C112 and C178). To avoid undesired coupling to the designed DNA handles during the coupling reactions, both C112 and C178 were mutated to serine. A codon-optimized gene encoding the cysteine-free TrmD with the desired restriction sites (5′ *Bam*HI and 3′ *Bgl*II and *Kpn*I) was custom synthesized (GeneScript). To avoid the inefficient coupling between the DNA handles and the first amino acid residue of wild-type TrmD, which was partially buried in the N-terminal subdomain, the 9^th^ amino acid residue phenylalanine was mutated to cysteine (F9C) using the megaprimer approach of site-directed mutagenesis.^41^ Following our well-established iterative molecular biology strategy,^42^ we constructed the gene TrmD_9,246_–(NuG2)_2_. The gene TrmD_9,246_–(NuG2)_2_ was then subcloned into a modified expression vector pQE80L–Cys, which carried a cassette allowing us to add one cysteine residue only at the C-terminus of the target protein, to build pQE80L/TrmD_9,246_–(NuG2)_2_–Cys. The full sequence of the engineered TrmD_9,246_–(NuG2)_2_–Cys is shown in the ESI.†

The gene encoding TrmD_45,128_ was constructed using the megaprimer approach. TrmD Δhelix_9,161_ and TrmD Δhelix_45,128_ were obtained by a further regular polymerase chain reaction (PCR) to remove the C-terminal α helical domain (residues 162–246). TrmD Δhelix_45,128_ and TrmD_45,128_ were subcloned into the pQE80L vector for protein expression. All of the constructed genes were confirmed by DNA sequencing.

The engineered protein was overexpressed in *Escherichia coli* strain DH5α at 37 °C in 250 mL 2.5% LB media with 100 mg L^−1^ antibiotic ampicillin. 1 mM isopropyl-β-d-1-thiogalactopyranoside (IPTG, Thermo Fisher Scientific, Waltham, MA) was added to induce protein overexpression at an optical density of around 0.8. The protein overexpression was allowed to continue for 4 hours at 37 °C. The bacterial cell pellets were harvested by centrifugation at 5000 rpm at 4 °C for 10 minutes and resuspended in 10 mL phosphate buffered saline (PBS, 10 mM, pH 7.4) buffer. 10 μL protease inhibitor cocktail (SIGMA-ALDRICH, St. Louis, MO), 50 μL 100 mg mL^−1^ lysozyme from egg white (SIGMA-ALDRICH, St. Louis, MO), 1 mL 10% (w/v) Triton X-100 (VWR, Tualatin, OR), 50 μL 1 mg mL^−1^ DNase I (SIGMA-ALDRICH, St. Louis, MO) and 50 μL 1 mg mL^−1^ RNase A (Bio Basic Canada Inc, Markham, ON) were added for cell lysis. The lysis reaction was kept at 4 °C for 40 minutes. The supernatant containing the target protein was isolated by centrifugation at 10 000 rpm at 4 °C for 1 hour, and the protein was purified using a Co^2+^ affinity column with a TALON His-tag purification kit (TaKaRa Bio USA Inc, Mountain View, CA). The protein was eluted and stored in elution buffer (10 mM PBS, 300 mM NaCl, 250 mM imidazole). The purified protein was at a concentration of ∼0.5 mg mL^−1^ and stored at −20 °C.

### Preparation of DNA handles and DNA–protein chimera

DNA handles were prepared *via* the method described previously.^34,40,43^ Two DNA handles were synthesized *via* regular PCR amplification by using pGEMEX-1 plasmid as the template and the modified primers purchased from Integrated DNA Technologies (IDT Inc, San Jose, CA), as reported previously.^34^ A QIAquick PCR purification kit (QIAGEN, Germantown, MD) was used to purify the PCR products followed by PCR amplification. The length of the DNA handles is 802 bp (biotin–DNA–maleimide) and 558 bp (digoxigenin–DNA–maleimide), which corresponds to the contour length of 273 nm and 190 nm, respectively. Then the DNA handles were allowed to react with 4-(*N*-maleimidomethyl) cyclohexanecarboxylic acid *N*-hydroxysuccinimide ester (SMCC, SIGMA-ALDRICH, St. Louis, MO) overnight. The freshly expressed proteins were reduced with 1 mM tris(2-carboxyethyl) phosphine (TCEP, SIGMA-ALDRICH, St. Louis, MO) for 1 hour and purified using Zeba desalting columns (7k MW, Thermo Fisher Scientific, Waltham, MA) to remove the extra TCEP. The reduced proteins were diluted to ∼3 μM using Tris buffer (20 mM Tris, 150 mM NaCl, pH 7.4) and 1 μL of the diluted protein was allowed to react with 1 μL of 3 μM prepared DNA handles at room temperature overnight. The DNA–protein chimera was diluted to ∼10 nM and stored at −80 °C.

### Optical tweezers experiments

The optical tweezers experiments were carried out on a MiniTweezers setup.^44,45^ 1 μL of streptavidin-coated polystyrene beads (1% w/v 1 μm, Spherotech Inc, Lake Forest, IL) was diluted using 3 mL Tris buffer and injected into the fluid chamber *via* a syringe. Subsequently, a single streptavidin-coated bead was captured by a laser beam and moved onto the tip of a glass pipette. The bead was fixed tightly by applying a vacuum through the pipette. 1 μL of the 10 nM DNA–protein chimera was added to 4 μL of anti-digoxigenin-coated polystyrene beads (0.5% w/v, 2 μm, Spherotech Inc, Lake Forest, IL) for coupling. The coupling reaction was kept at room temperature for 30 minutes. The beads coupled with the DNA–protein chimera on its surface were also diluted with 3 mL Tris buffer and injected into the fluid chamber. A single anti-digoxigenin-coated bead was then trapped by a laser beam and brought into contact with the streptavidin-coated bead to establish the dumbbell for stretching and relaxation (Fig. 1B).

Stretching the protein–DNA chimera resulted in force–distance curves, in which the distance contains the contribution of the extension of the protein–DNA construct as well as the compliance of the optical trap. The MiniTweezers setup is a nonlinear optical trap, and its stiffness varies with the stretching force.^44,45^ Therefore, force–distance curves measured using the MiniTweezers setup cannot be directly converted into force–extension curves of the protein–DNA construct itself. As such, the force–distance curves cannot be directly fitted to the worm-like-chain (WLC) model of polymer elasticity to measure the contour length increment (Δ*L*_c_) of protein unfolding. For a given unfolding event with a contour length increment of Δ*L*_c_ in the force–distance curve, the length increase at a given force *F* gives the extension *E* of the unfolded polypeptide chain of a contour length of Δ*L*_c_. By fitting the measured force–extension relationship to the WLC model, we can measure the persistence length and Δ*L*_c_ of the unfolded polypeptide chain being released from the unfolding.^33,40,46^

### Extracting the protein unfolding/folding kinetics

A direct and model-free calculation method proposed by Oesterhelt *et al.*^47^ was used to measure the force-dependent two-state unfolding/folding rate constants of proteins. The force–distance curves, generated by stretching (or relaxing) the target protein at a constant velocity, were divided into small time windows (Δ*t*) and the force can be regarded as constant within the time window. The unfolding/folding rate constants at force *F* can be calculated as *k*(*F*) = *N*(*F*)/*M*(*F*) × Δ*t*, where *N*(*F*) is the total number of unfolding/folding events observed at force *F* and *M*(*F*) is the total number of time windows at force *F*. In our experiments, we used a bin of 1 pN in force, which will give rise to a Δ*t* of 0.2–0.3 s. This time window was small enough to allow the use of the Oesterhelt method to extract rate constants with good accuracy.

Force-dependency of the unfolding rate constants of short-lived intermediate states was measured directly by a single exponential fitting to the dwell-time distribution of the intermediate states.^48^ The relationship of probability density *vs.* dwell-time can be described as Pd(*t*) = *α*(*F*)exp(*α*(*F*) × *t*), where *α*(*F*) is the unfolding rate constant under force *F*, *t* is the dwell-time of short-lived intermediate states and Pd(*t*) is the probability density for a specific dwell-time.

The force-dependency of unfolding/folding rate constants was subsequently fitted using the Bell–Evans model, to extract the intrinsic protein unfolding/folding kinetics at zero force.^49,50^ The Bell–Evans model can be described as *α*(*F*) = *α*_0_ exp(*F*Δ*x*_u_/*k*_B_*T*) and *β*(*F*) = *β*_0_ exp(−*F*Δ*x*_f_/*k*_B_*T*), where *k*_B_ is the Boltzmann constant, *T* is the temperature in Kelvin, *α*(*F*) (or *β*(*F*)) is the unfolding (or folding) rate constant under force *F*, and *α*_0_ (or *β*_0_) is the intrinsic unfolding (or folding) rate constant at 0 pN. Δ*x*_u_ (or Δ*x*_f_) is the distance from the native protein to transition state (or from the unfolded to transition state).

A standard double-pulse protocol^42^ was applied to measure the knotting kinetics of mutant TrmD Δhelix_45,128_. The protein was first untied under stretching, and then quickly relaxed to its initial length. After a time delay Δ*t*, a second stretching was applied to the molecule to check if the knotting was achieved again within the time Δ*t*. Protein knotting probability was dependent on the time delay for protein refolding and can be expressed as *P*(*t*) = 1 − exp(*β*_0_ × *t*), where *β*_0_ is the intrinsic protein knotting rate constant.

## Results

### Stretching TrmD leads to the mechanical unfolding of TrmD and the tightening of the trefoil knot

To investigate the mechanical unfolding of the trefoil knotted protein TrmD using OT, we stretched TrmD from its C-terminus (residue 246) and residue 9 using the TrmD variant Phe9Cys (termed TrmD_9,246_) of which the more solvent exposed residue 9 (than the N-terminus) (Fig. 1) helped improve the coupling efficiency of DNA handles to the protein. We then engineered the dsDNA–protein chimera, DNA–TrmD_9,246_–(NuG2)_2_–DNA, by coupling doubled stranded DNA handles to the construct TrmD_9,246_–(NuG2)_2_*via* thiol-maleimide coupling chemistry.^33,34,40,43^ In this DNA–protein construct, the well-characterized NuG2 domains serve as a fingerprint for identifying single-molecule stretching events.^34,43^

**Fig. 2:**
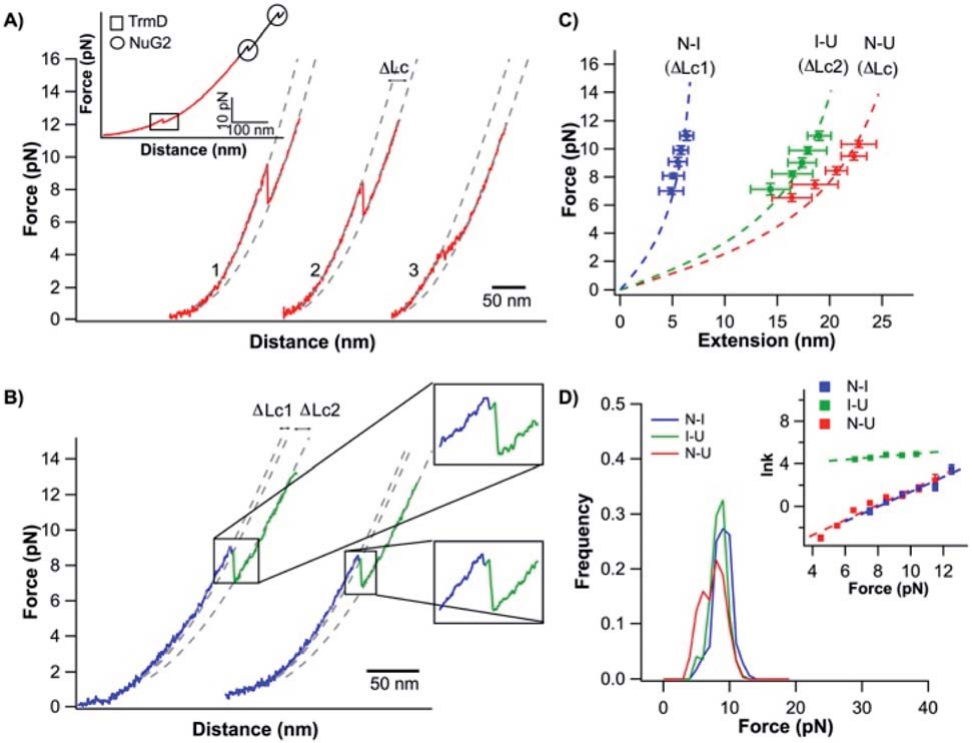
Stretching TrmD_9,246_ to a tightened trefoil knot involves two parallel pathways. (A) Representative force–distance curves showing two-state unfolding of TrmD_9,246_. The pulling speed was 50 nm s^−1^. Inset: representative force–distance curves showing the unfolding of full-length TrmD_9,246_–(NuG2)_2_. The mechanical unfolding events of the fingerprint domains NuG2 are colored in black and indicated with circles, and the unfolding events of TrmD_9,246_ are indicated with squares. (B) Representative force–distance curves of TrmD_9,246_*via* the three-state unfolding pathway. The pulling speed was 50 nm s^−1^. The zoom-in view showed that the protein unfolds *via* an intermediate state. The protein unfolding from N → I and I → U is colored in blue and green, respectively. The dashed lines in (A) and (B) are pseudo WLC fits to the force–distance curves. The fits were primarily used for identification of various states (folded, intermediate and unfolded state) of protein in the force–distance curves, and the fitting parameters were not physically meaningful. (C) Force–extension relationships of TrmD_9,246_. Extension is the length increase at a given force upon unfolding. Error bars correspond to the standard deviation of the data measured from different molecules. Dashed lines are WLC fits to the force–extension data measured for N → I (blue), I → U (green) and N → U (red), respectively. The WLC fits yielded a persistence length of 0.8 nm and a Δ*L*_c1_ of 9.8 ± 0.4 nm, Δ*L*_c2_ of 28.9 ± 1.0 nm and Δ*L*_c_ of 36.8 ± 1.8 nm, respectively. (D) Unfolding force histograms of TrmD_9,246_ at a pulling speed of 50 nm s^−1^. Inset: force-dependency of unfolding rate constants measured by the model-free method proposed by Oesterhelt *et al.* Fitting to the Bell–Evans model yielded the intrinsic unfolding rate constant *α*_0_ and the unfolding distance Δ*x*_u_, as summarized in Table S1.†

Stretching TrmD_9,246_–(NuG2)_2_ resulted in force–distance curves with the characteristic sawtooth-like appearance (Fig. 2A, inset). Three unfolding events were observed prior to the B–S overstretching transition of the dsDNA handles at ∼65 pN. Two unfolding events occurred at higher forces (typically higher than 30 pN) with a Δ*L*_c_ of 18 nm (colored in black), corresponding to the mechanical unfolding of the fingerprint NuG2 domains. Thus, the third unfolding event at ∼10 pN can be readily attributed to the unfolding of TrmD (colored in red). Since TrmD unfolds at forces that are significantly lower than those for NuG2, subsequent stretching of TrmD was limited to ∼15 pN so that only TrmD was unfolded and NuG2 domains remained folded in the experiment (Fig. 2A and B).

The mechanical unfolding of TrmD_9,246_ was observed to follow bifurcated pathways, involving both two-state and three-state pathways. Fig. 2A shows typical force–distance curves of TrmD following the two-state unfolding pathway. Fitting the force–extension relationships to the WLC model of polymer elasticity gave a contour length increment Δ*L*_c_ of 36.8 nm ± 1.8 nm upon the unfolding of TrmD (Fig. 2C). TrmD is 246 amino acid residues long, but only 238 residues were involved in the pulling experiments of TrmD_9,246_. The complete unfolding of TrmD_9,246_ would lead to a Δ*L*_c_ of ∼80 nm (238 aa × 0.36 nm per aa − 5.4 nm = 80.3 nm, where 5.4 nm is the distance between residues 9 and 246) if there was no knotted conformation. It is evident that the calculated Δ*L*_c_ is significantly larger than the experimentally measured one, suggesting that part of TrmD_9,246_ may have unfolded at low forces, which are below the detection limit of the MiniTweezers.

It is of note that TrmD contains a large C-terminal α-helical domain (residues 133 to 246). The α-helix structure is mechanically labile and often unfolds at very low forces, resulting in the missing of a clear mechanical unfolding signature in single-molecule force spectroscopy experiments.^16,51^ We speculated that the unfolding of the labile α-helix (including the C-terminal α-helical domain and the α-helix in the knot core (residues 133–161)) would account for the missing Δ*L*_c_ in TrmD. To verify this hypothesis, we engineered an α-helix truncation variant TrmD Δhelix (residues 9–161), in which residues 162–246 were deleted. Stretching TrmD Δhelix_9,161_ resulted in a Δ*L*_c_ of ∼37 nm, identical to that of wt TrmD (Fig. S1†), suggesting that indeed the unfolding of the C-terminal α-helix occurred at low forces and in a gradual fashion and did not result in a clear unfolding signature in the force–distance curves.

If the α-helix in the knot core (residues 133–161) also unfolds at low forces similar to the C-terminal α-helical domain, the unfolding of the rest of the folded protein structure (residues 9 to 132) would yield a Δ*L*_c_ of 42.7 nm (124 aa × 0.36 nm per aa − 1.9 nm = 42.7 nm, where 1.9 nm is the distance between Cys9 and Ser132). This Δ*L*_c_ of 42.7 nm is about 6 nm longer than the experimentally measured Δ*L*_c_ of TrmD_9,246_. Distinct from the unfolding of NuG2, the unfolding of TrmD_9,246_ would result in a tightened trefoil knot present within the unstructured polypeptide chain. Thus, the “missing Δ*L*_c_” of ∼6 nm, corresponding to ∼17 amino acid residues, can be readily attributed to the formation of the tightened trefoil knot. This result is in good agreement with the size of the protein trefoil knot measured previously in single-molecule force spectroscopy experiments,^8,32^ and suggested that TrmD can be mechanically stretched into an unfolded conformation with a tightened trefoil knot.

TrmD_9,246_ unfolds *via* a two-state pathway, with an average unfolding force of ∼8 pN at a pulling speed of 50 nm s^−1^ (Fig. 2D and Table S1†). Using the method proposed by Oesterhelt *et al.*,^47^ we measured the unfolding rate constant as a function of the stretching force (inset, Fig. 2D). Fitting the experimental data to the Bell–Evans model^49,50^ gave an intrinsic unfolding rate constant *α*_0_ of (3.8 ± 0.5) × 10^−3^ s^−1^ at zero force and an unfolding distance between the native state and transition state Δ*x*_u_ of 2.9 ± 0.3 nm.

### The mechanical unfolding of TrmD involved a short-lived intermediate state

In our OT experiments, about 68% of TrmD_9,246_ followed an apparent two-state unfolding pathway. A small portion (∼32%) of TrmD_9,246_ was observed to unfold *via* a three-state pathway, involving a short-lived unfolding intermediate state (Fig. 2B). WLC fits to the extension at different forces yielded a Δ*L*_c1_ of 9.8 ± 0.4 nm and a Δ*L*_c2_ of 28.9 ± 1.0 nm, respectively (N → I and I → U, Fig. 2C). The sum of Δ*L*_c1_ and Δ*L*_c2_ equals 38.7 nm, in good agreement with the Δ*L*_c_ yielded from two-state unfolding pathway. These results suggested that the mechanical unfolding pathways of TrmD are bifurcated,^52,53^ and the three-state pathway involves a well-defined intermediate state during the mechanical unfolding pathway.

In the three-state pathway, the first unfolding event (from the native to the intermediate state) occurred at an unfolding force of 9.5 ± 1.3 pN. And the second unfolding event (from the intermediate state to the unfolded state) occurred at 8.9 ± 1.3 pN (Fig. 2D and Table S1†). It is of note that the unfolding intermediate state was short-lived in the range of force we measured (7–10 pN), and the lifetime, with an average of ∼0.02 s, was not sensitive to the stretching force. Fitting the force-dependency of the unfolding rate of the three-state unfolding to the Bell–Evans model yielded an intrinsic unfolding rate constant *α*_0_ of (3.6 ± 0.6) × 10^−3^ s^−1^ (N → I) and (3.6 ± 0.3) × 10^1^ s^−1^ (I → U) (inset, Fig. 2D and Table S1†), respectively. The unfolding rate constant for I → U was four orders of magnitude larger than that for N → I, suggesting that the intermediate state was rather mechanically unstable and can be unfolded rapidly once the intermediate state is formed.

It is of note that the unfolding kinetics parameters for N → I are similar to those of N → U. This similarity together with the short life time of the intermediate state gives rise to the possibility that the observed two-state pathway is just a special case of the three-state pathway, in which, due to the insufficient time resolution, the intermediate state was not resolved. Since our MiniTweezers setup has a temporal resolution of ∼0.1 ms at a force of ∼10 pN, we carried out experiments at a higher sampling frequency (30 kHz) to gain a better resolution of the unfolding events. Our data at higher sampling frequency only showed a small increase of the observed three-state unfolding events (∼30% at 1 kHz *vs.* ∼35% at 30 kHz). This result suggested that most of the two-state unfolding events were likely not due to the insufficient time resolution, and the two-state and three-state pathways are more likely to be two different pathways. However, the difference between the two unfolding pathways is small.

Nevertheless, it is important to point out that the observed unfolding intermediate state and the bifurcation of the unfolding pathways are not associated with the trefoil knot, but a property of the folded protein structure itself.

### The compaction of the trefoil knot

It is of interest to note that when the mechanical unfolding of TrmD_9,246_ occurred at ∼9 pN, the force–distance curve of the unfolded TrmD largely followed the typical WLC behavior of an unfolded polypeptide chain (Fig. 2A, curves 1 and 2). However, if the unfolding occurred at lower forces (<6 pN) (Fig. 2A, curve 3, and Fig. S2†), the force–distance curve of the unfolded TrmD showed a clear deviation from the WLC behavior of an unfolded polypeptide chain (grey dotted line). This behavior suggests that at lower forces, TrmD_9,246_ first unfolded into a state that is of a shorter *L*_c_, and further stretching caused the lengthening of the unfolded polypeptide chain until its length is the same as that of the unfolded TrmD_9,246_ with a tightened knot (of the size of 6 nm). This behavior can be explained by the compaction of the trefoil knot by the stretching force as schematically shown in Fig. S2.† At forces higher than 7 pN, the force–distance curve returned to the normal WLC behavior of an unfolded polypeptide chain, suggesting that the trefoil knot had been stretched to a tightened knot at a force of ∼6–7 pN. Further compaction/tightening would require higher forces. Similar compaction behavior was observed for a 5_2_ knot in the pulling experiments of UCH-L1.^8^

### The unfolded and knotted TrmD can refold back to its native state upon relaxation

**Fig. 3:**
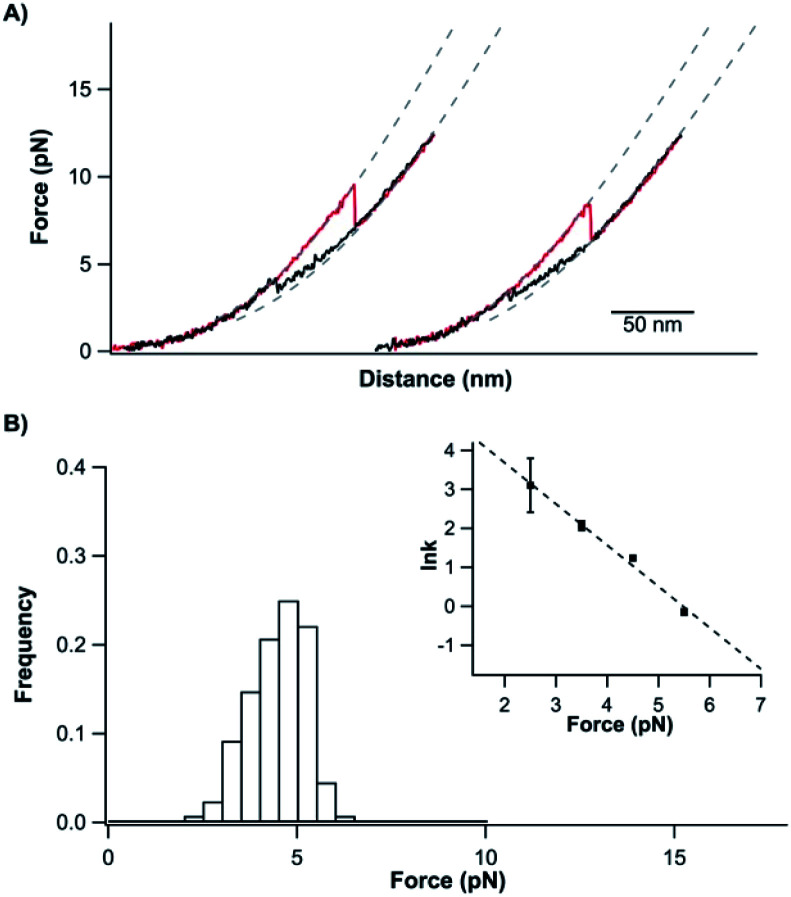
Unfolded and tightened TrmD_9,246_ can fold back to its native state. (A) Representative stretching–relaxation curves of TrmD_9,246_ at a pulling speed of 50 nm s^−1^. Consecutive stretching and relaxing curves revealed that the tightened protein was able to fold back to the native state. During relaxation, the force–distance relationship of TrmD deviated from the typical behaviour of a DNA–protein chimera starting from ∼6 pN (as indicated by the dotted pseudo WLC fits). The protein eventually folded back into its native state *via* a quasi-two-state mechanism at ∼4 pN. (B) The force histograms of the “quasi-two-state” folding of TrmD_9,246_. Inset: force-dependency of the folding rate constants measured using the Oesterhelt method. The dotted line is the fitting using the Bell–Evans model. Fitting parameters *β*_0_ and Δ*x*_f_ are shown in Table S1.†

After its mechanical unfolding, TrmD_9,246_ was converted to an unfolded polypeptide with a tightened trefoil knot. To investigate whether the unfolded TrmD with a tightened trefoil knot can refold back to its native state, we relaxed the unfolded polypeptide to allow protein refolding. As shown in Fig. 3A, during the relaxation process (curves colored in black), the protein was observed to refold, characterized by a force jumping event at ∼4 pN and a length shortening to the original length of TrmD_9,246_. Subsequent stretching led to an identical signature of the unfolding of the knotted protein TrmD_9,246_, suggesting that the relaxation had resulted in a successful refolding of the native conformation of TrmD_9,246_.

However, the folding behavior of TrmD_9,246_ is distinct from that of a typical two-state folding event. During the relaxation/refolding process of a typical two-state folder, such as NuG2 (Fig. S3†), the unfolded polypeptide chain followed the typical WLC behavior until the refolding event occurs. In contrast, during the relaxation of the unfolded TrmD_9,246_, the force–distance relationship of the unfolded TrmD_9,246_ polypeptide chain showed a clear deviation from that of WLC when the force was lower than ∼7 pN. When the force reached ∼4 pN, the unfolded TrmD refolded, as evidenced by a sudden force jump event. This behavior seemed to be the reverse of the compaction process of the trefoil knot observed during unfolding (Fig. S2†), suggesting that the global folding of the knotted TrmD_9,246_ may proceed with the loosening process of the tightened trefoil knot. Moreover, the reversibility of the compaction and loosening processes and their deviation from the WLC model suggested that elastic energy was stored in the polypeptide chain during the knot compaction process, which was released during the loosening process.

The “quasi-two-state” folding forces of TrmD_9,246_ showed a narrow distribution with an average force of ∼4.5 pN at a pulling speed of 50 nm s^−1^ (Fig. 3B and Table S1†). Moreover, fitting the force-dependent folding rate constants to the Bell–Evans model gave a folding rate constant at zero force of (3.3 ± 0.3) × 10^2^ s^−1^ (inset, Fig. 3B). These results indicated that the folding of TrmD from its unfolded state with the pre-formed knot is fast and robust.

### Untying and tying the knotted structure of TrmD by using optical tweezers

**Fig. 4:**
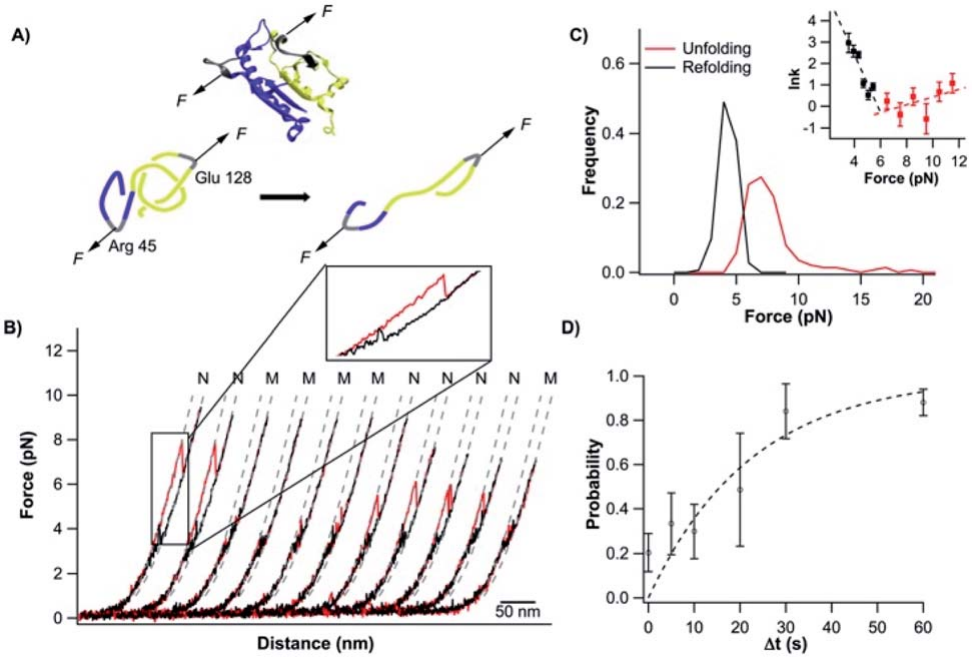
Untying and tying the knotting loop of TrmD Δhelix_45,128_. (A) Schematics of stretching protein TrmD Δhelix_45,128_. The stretching sites are colored in grey. (B) Representative stretching–relaxation curves of TrmD Δhelix_45,128_ showing distinct unfolding behavior of the same molecule. Relaxation of the unfolded protein led to a “refolding” event at ∼4 pN. However, it remained unknown whether the protein folded into its native state with the trefoil knot. In the subsequent stretching force–distance curves, either a typical two-state unfolding at a force of ∼7 pN or a “hump” feature was observed. (C) Histograms of unfolding and “refolding” forces of TrmD Δhelix_45,128_ at a pulling speed of 50 nm s^−1^. Inset: force-dependency of the unfolding/folding rate constants. Dotted lines are fits of the experimental data to the Bell–Evans model. (D) Relationship of the knotting probability *versus* Δ*t* at zero force. The dotted line is the fit of the experimental data to the first order rate law, yielding a knotting rate constant of (3.8 ± 1.0) × 10^−2^ s^−1^.

Having characterized the mechanical unfolding of TrmD_9,246_ and the folding of unfolded and knotted TrmD_9,246_, we then investigated the possibility of unfolding TrmD and untying its trefoil knot. The trefoil knot in TrmD is very deep, and even removing 79 residues from either N- or C-termini will not destroy the trefoil knot. This deep knot is difficult to untie in a force spectroscopy experiment. In order to untie the trefoil knot in TrmD, we used the helix truncation variant TrmD Δhelix, in which the depth of the trefoil knot is significantly reduced. Residues Arg45 and Asp128 were mutated to cysteines to allow TrmD to be stretched from these two residues (this construct is termed TrmD Δhelix_45,128_) (Fig. 4A).

Stretching TrmD Δhelix_45,128_ resulted in the typical force–distance curves shown in Fig. 4B. The unfolding of TrmD Δhelix_45,128_ largely follows a two-state unfolding pathway with an average unfolding force of ∼8 pN at a pulling speed of 50 nm s^−1^. Fitting the WLC model of polymer elasticity to the extension of Δhelix_45,128_ led to a Δ*L*_c_ of 25.6 ± 0.7 nm upon the unfolding of TrmD Δhelix_45,128_ (Fig. S4†). When stretched from residues 45 and 128, 84 residues were involved. The complete unfolding of TrmD Δhelix followed by the untying of the trefoil knot would result in a Δ*L*_c_ of 26.9 nm (84 aa × 0.36 nm per aa − 3.3 nm = 26.9 nm, where 3.3 nm is the distance between C45 and C128), in excellent agreement with the experimentally measured Δ*L*_c_. This result strongly indicated that stretching the TrmD Δhelix from residues 45 and 128 resulted in the mechanical unfolding of the TrmD Δhelix and untying of its trefoil knot, providing a unique opportunity to investigate the knotting and refolding process of TrmD.

TrmD Δhelix_45,128_ unfolds *via* a two-state pathway with an average unfolding force of ∼8 pN at a pulling speed of 50 nm s^−1^ (Fig. 4C and Table s1†). Fitting the force-dependent unfolding rate constants to the Bell–Evans model yields an intrinsic unfolding rate constant *α*_0_ of (2.5 ± 2.2) × 10^−1^ s^−1^ (Fig. 4C, inset). After the TrmD Δhelix_45,128_ had been unfolded and untied, the unfolded and unknotted polypeptide chain was relaxed to zero force to allow the folding/knotting of TrmD. During the relaxation process, a “refolding” event was often observed at ∼4 pN, leading to a “refolded” state that is of similar length to the native Δhelix_45,128_ (Fig. 4B). The average “refolding” force is ∼4 pN and the intrinsic “refolding” rate constant was estimated to be (2.5 ± 0.4) × 10^3^ s^−1^ (Fig. 4C, inset). However, it was unclear whether the trefoil knot had reformed in this “refolded” state. To check this, we stretched the “refolded” state in the next cycle. In about 20% of the traces, a clear unfolding event occurring at ∼7 pN was observed (Fig. 4B, curves marked by “N”). The unfolding signatures of these events were the same as those of the native TrmD Δhelix with the trefoil knot, suggesting that in these “refolded” molecules, the trefoil knot had been retied. However, in 80% of the traces, the unfolding occurred at ∼4 pN, resulting in a hump-like feature (Fig. 4B, curves marked by “M”). This unfolding signature is different from that of unfolding of the native TrmD Δhelix_45,128_, suggesting that in these events, TrmD Δhelix_45,128_ folded into a non-native or intermediate state that is mechanically labile, and the trefoil knot likely had not reformed. Therefore, the observed “refolding” at ∼4 pN for TrmD Δhelix_45,128_ did not reflect the true refolding of the native trefoil TrmD. Instead, it is likely that such “refolding” events reflected the folding into an unknotted, non-native state or intermediate state.

The knotted conformation of TrmD is an important structural feature of the enzyme. Since the mechanical stretching of TrmD Δhelix_45,128_ involved untying the trefoil knot by pulling the C-terminus of the protein threading out of the knot core, the correct folding of TrmD must involve tying the C-terminal knot tail into the knot core. It has been shown that knotting is the rate-limiting step in the folding of some knotted proteins.^8,54^ To investigate if knotting is the rate limiting step for TrmD Δhelix_45,128_, we used OT to investigate the folding behavior of wt TrmD by stretching wt TrmD from its residues 45/128 (TrmD_45,128_).

**Fig. 5:**
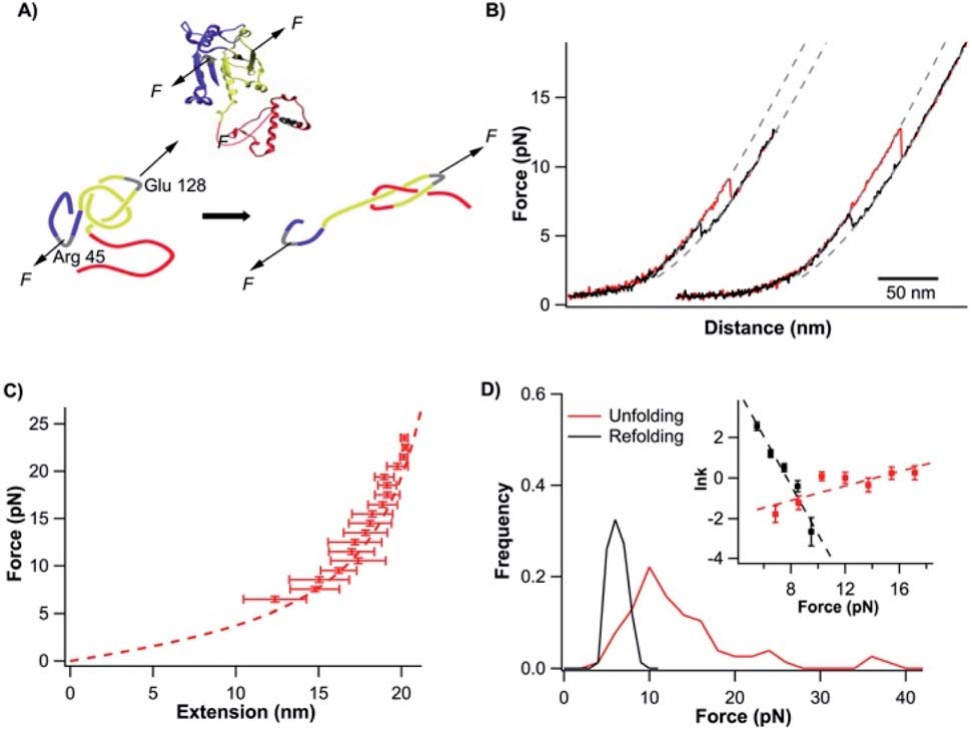
Stretching the protein TrmD_45,128_ does not untie the knotted conformation. (A) Schematics of stretching protein TrmD_45,128_. The stretching site is colored in grey. (B) Representative force–distance curves of TrmD_45,128_. Consecutive stretching–relaxation curves revealed that the protein can always fold back to its native state. (C) WLC fits to the force–extension relationship of TrmD_45,128_ gave a persistence length of 0.8 nm and Δ*L*_c_ of 27.6 nm. (D) Unfolding and refolding force histograms of TrmD_45,128_ at a pulling speed of 50 nm s^−1^. The inset shows the force-dependency of the unfolding and folding rate constants. Fitting the experimental data to the Bell–Evans model yielded the intrinsic unfolding/folding rate constants, *α*_0_ = (7.9 ± 2.0) × 10^−2^ s^−1^ and *β*_0_ = (1.0 ± 0.1) × 10^4^ s^−1^, as well as the unfolding/folding distance, Δ*x*_u_ = 0.7 ± 0.2 nm and Δ*x*_f_ = 4.9 ± 0.6 nm.

To untie a protein knot, the length of the polypeptide chain between the two stretching points in the protein knot structure should be much longer than the length of the knot tail to be pulled out of the knot. For the pulling geometry of TrmD_45,128_, TrmD can be unfolded but its deep trefoil knot cannot be untied due to the long C-terminal knot tail (residues 162–246). Our OT experiments showed that the unfolding of TrmD_45,128_ occurred at higher forces than those for wt TrmD, but displayed a Δ*L*_c_ of 27.6 ± 0.7 nm (Fig. 5B and C), which is the same as that of TrmD Δhelix_45,128_. But the folding of TrmD_45,128_ was drastically different from that of TrmD Δhelix_45,128_. The folding of TrmD_45,128_ occurred in a two-state fashion at ∼6 pN (Fig. 5D). Consecutive stretching–relaxation cycles showed that TrmD_45,128_ could always fold correctly back to its native state, and no misfolding was observed. Kinetics analysis revealed a high folding rate constant at zero force (Fig. 5D, inset), indicating that TrmD_45,128_ can fold rapidly in the presence of a pre-formed knotted structure.

The comparison of the folding behavior of TrmD_45,128_ and TrmD Δhelix_45,128_ clearly indicated the critical importance of the knotting step in the folding of TrmD. To determine the knotting kinetics of TrmD Δhelix_45,128_, we used a double-pulse protocol in the refolding experiment.^42^ In the first pulse, the correctly folded and knotted TrmD Δhelix_45,128_ was stretched to unfold the protein and untie the trefoil knot, and then the unfolded and untied polypeptide chain was allowed to relax to zero force and held at zero force for a period of time Δ*t*. In the second pulse, the protein was stretched again. The occurrence of the unfolding signatures of the correctly folded and knotted TrmD Δhelix_45,128_ would indicate the successful knotting and folding of the protein. As shown in Fig. 4D, the percentage of successful knotting and folding events of TrmD Δhelix_45,128_ increased with the increasing of waiting time Δ*t*. Assuming a first-order kinetics, we estimated a rate of knotting of TrmD Δhelix of 3.8 ± 1.0 × 10^−2^ s^−1^. This rate constant is considerably smaller than the folding rate of TrmD with a pre-formed knotting loop, suggesting that the protein knotting is indeed the rate-limiting step for the folding of the TrmD Δhelix. Considering the depth of the trefoil knot in wt TrmD, it is likely that the knotting step of wt TrmD is significantly slower than that of TrmD Δhelix, raising an interesting question if the folding of TrmD *in vivo* involves any molecular chaperone.

It is also important to note that the folding of the unfolded TrmD_45,128_ also showed significant difference from the folding behavior of the unfolded TrmD_9,246_. The force–distance relationship of the unfolded TrmD_45,128_ largely followed the WLC model, and no loosening process of the tightened trefoil knot was observed. This behavior can be readily understood by the unfolding behavior of TrmD_45,128_ schematically shown in Fig. 5A. In this pulling geometry, the stretching force will not untie the trefoil knot (as occurred for TrmD Δhelix_45,128_) due to the long C-terminal knot tail or tighten the trefoil knot (as occurred for wt TrmD_9,246_) after the unfolding of TrmD_45,128_. Instead, the stretching force acting on TrmD_45,128_ will only expand the trefoil knot structure, leading to the absence of a tightened knot conformation in the unfolded TrmD_45,128_. Thus, the folding of the unfolded TrmD_45,128_ does not involve the loosening up of the knot structure, but largely reflects the folding of the three dimensional structure of TrmD.

## Discussion

The deep trefoil knot structure is a common and unique feature of the SPOUT family of RNA methyltransferases (MTases), which catalyze the methylation of the base or ribose moiety of ribosomal RNA (rRNA) or transfer RNA (tRNA). TrmD is a conserved SPOUT MTase in bacteria and responsible for the methylation of tRNA.^26–31^ It was proposed that the trefoil knot in TrmD is critical for its function, as the trefoil knot is responsible for accommodating the adenosine moiety of the methyl donor of AdoMet in the methylation process by loosening and retightening of the trefoil knot.^27^ Thus, correct folding of TrmD into its knotted structure is crucial.

By using single-molecule OT, we have investigated the mechanical unfolding, tightening and untying of the trefoil knot, as well as the refolding of TrmD starting from its unfolded polypeptide with or without a pre-formed knot structure. Our results provided some new insights into the unfolding and folding mechanisms of TrmD.

After the mechanical unfolding of TrmD, we observed a mechanical compaction process of the trefoil knot at low forces (<6 pN), which led to the lengthening of contour length of the unfolded polypeptide chain and tightening of the trefoil knot. This is the first time that such a compaction process is observed in a simple trefoil knot. The fact that the compaction process was only observed at low forces suggested that the compaction process may have completed in the taut unfolded polypeptide chain when TrmD was unfolded at higher forces, as in curves (1) and (2) in Fig. 2A. Moreover, after the compaction process had completed, the length of the unfolded polypeptide chain remained largely unchanged upon further stretching, implying that the knot size remained largely constant. And further tightening and even “jamming” of the trefoil knot (*i.e.* shrinking the knot size) will require much higher forces than that the OT can apply (typically <100 pN) (Fig. S5†). Thus, the stretching of the trefoil knot in the OT experiments most likely only resulted in a limited further tightening.

When the force is lower than ∼7 pN during the relaxation process, the thermal energy is sufficient to loosen up the knot tightened by a force at least up to 50 pN (Fig. S5†) and the knot can then expand. The observed compaction and loosening of the trefoil knot at low forces revealed the dynamic nature and structural plasticity of the trefoil knot structure in TrmD. It is worth noting that this compaction and loosening of the trefoil knot occurs when TrmD is unfolded. Further experiments to demonstrate that similar processes can occur in the folded TrmD will be critical for a deeper understanding of the function mechanism of TrmD, as crystallography studies suggested that loosening and tightening of the knot are critical for accommodating the adenosine moiety of the methyl donor of AdoMet in the methylation process by TrmD.^27^

It is also of note that such a compaction and loosening process of the trefoil knot was not observed in a trefoil knot prepared from UCH-L1 in a previous OT study.^8^ Whether such a compaction and loosening process is related to the protein sequence and/or residual secondary structure remains to be elucidated.

Our results showed that the folding of TrmD with a pre-formed knot is fast and robust, and the knotting is the rate-limiting step for the overall folding of TrmD. These findings complement the previous results obtained on knotted proteins (a 5_2_ knotted UCH-L1 and trefoil-knotted methyltransferases YibK and YbeA),^8,54^ and raise the question if this feature is common among all knotted proteins.

It is important to point out that, due to the pulling geometry in TrmD_45,128_, the knotting can only occur by threading the C-terminal end in our OT experiment. In comparison, the depth of the trefoil knot in wild type TrmD prevented us from preparing an untied wild type TrmD, and thus it is not possible to directly study the knotting and folding process of wt TrmD using OT. Given the depth of the trefoil knot in wt TrmD, it is most likely that the folding of TrmD *in vivo* is different from what we observed in TrmD_45,128_. More studies, likely involving molecular chaperones or folding on ribosomes, will be needed to elucidate the folding mechanism of wt TrmD *in vivo*.

## Conclusions

Combining optical tweezers and protein engineering techniques, we investigated the mechanical unfolding and folding behavior of a knotted protein TrmD. Our results showed that TrmD can be mechanically unfolded along a direction defined by two specific residues. Depending on the pulling geometry, the deep trefoil knot of TrmD can take different conformations. It can be tightened, untied, or expanded in the unfolded state by the stretching force. After the mechanical unfolding of TrmD, a compaction process of the trefoil knot was observed at low forces. The folding of TrmD strictly depends on the conformation of the unfolded TrmD. If the unfolded TrmD contains a pre-formed knot (tightened or not), the folding of TrmD is fast and robust. If the unfolded TrmD does not contain a pre-formed knot, the knotting is the rate-limiting step for the folding. Our study provided detailed mechanistic insights into the folding and unfolding of TrmD, and revealed the intricate interplay between the folding and knotting process of TrmD.

## Conflicts of interest

There are no conflicts to declare.

## Supplementary Material

SC-011-D0SC02796K-s001
