# Supplementary material for: Mechanically tightening, untying and retying a protein trefoil knot by single-molecule force spectroscopy
Source: Chem Sci. 2020 Oct 19;11(46):12512–21. doi: 10.1039/d0sc02796k (PMC8162576; doi:10.1039/d0sc02796k)
Supplement: SC-011-D0SC02796K-s001 [file SC-011-D0SC02796K-s001.pdf]

Electronic Supplementary Material (ESI) for Chemical Science.
This journal is © The Royal Society of Chemistry 2020

Mechanically Tightening, Untying and Retying a Protein Trefoil Knot by Single Molecule Force Spectroscopy

Han Wang and Hongbin Li*

Electronic Supplementary Information

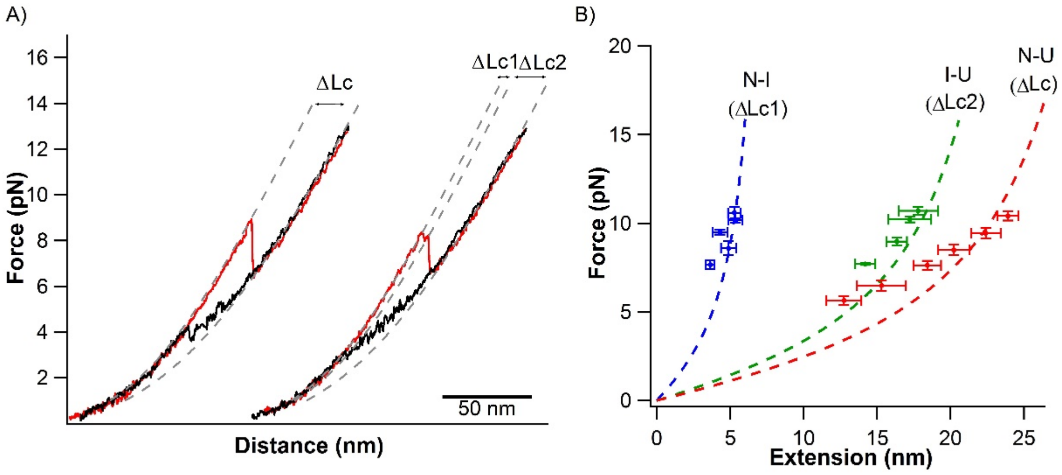

Figure S1. Mechanical unfolding and folding of TrmD Δhelix$_{9,161}$. A) Representative force-distance curves showing the unfolding and folding signatures were identical to those of TrmD$_{9,246}$. Both two-state and three-state unfolding were observed for protein unfolding. Upon relaxation, the tightened trefoil knot followed a quasi-two state pathway to the native state. B) Force-extension relationship of TrmD Δhelix$_{9,161}$. The WLC fits measured a persistence length of 0.8 nm and ΔLc1 of 8.7±0.7 nm, ΔLc2 of 29.7±1.6 nm and ΔLc of 37.5±2.4 nm, respectively.

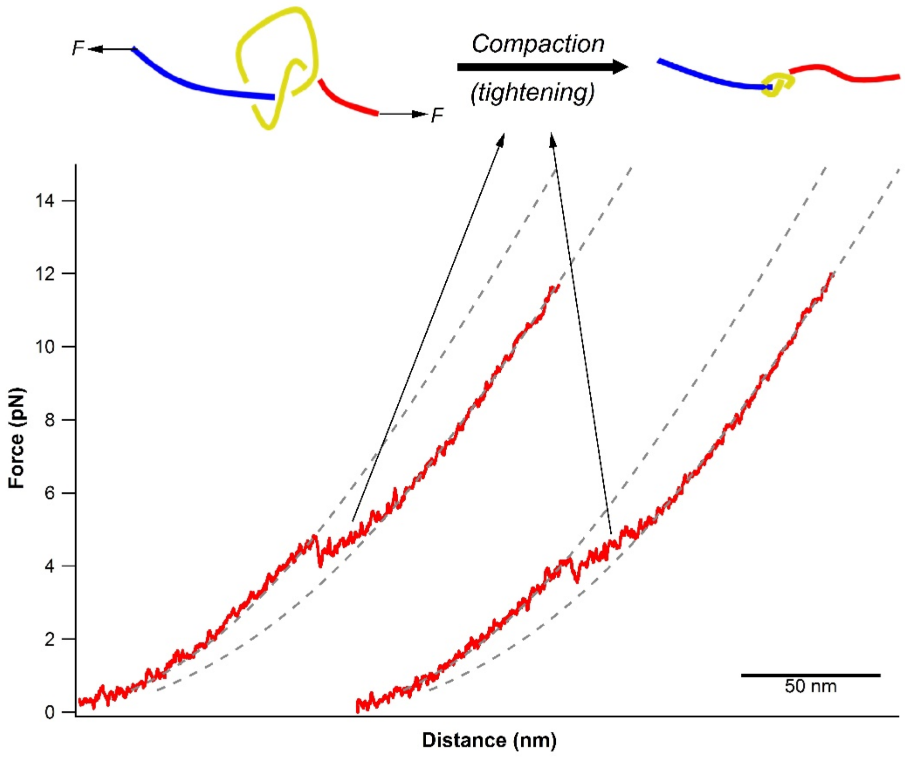

Figure S2. Representative force-distance curves showed the mechanical unfolding of $TrmD_{9,246}$ at low forces. A clear deviation from a typical WLC behavior of an unfolded polypeptide chain can be observed at the early stage. Further stretching leads to the polypeptide lengthening until the force-distance curve returns to follow the WLC behavior. This polypeptide lengthening process corresponds to the compaction and tightening of the knot structure within the protein, which is shown in the schematics on the top.

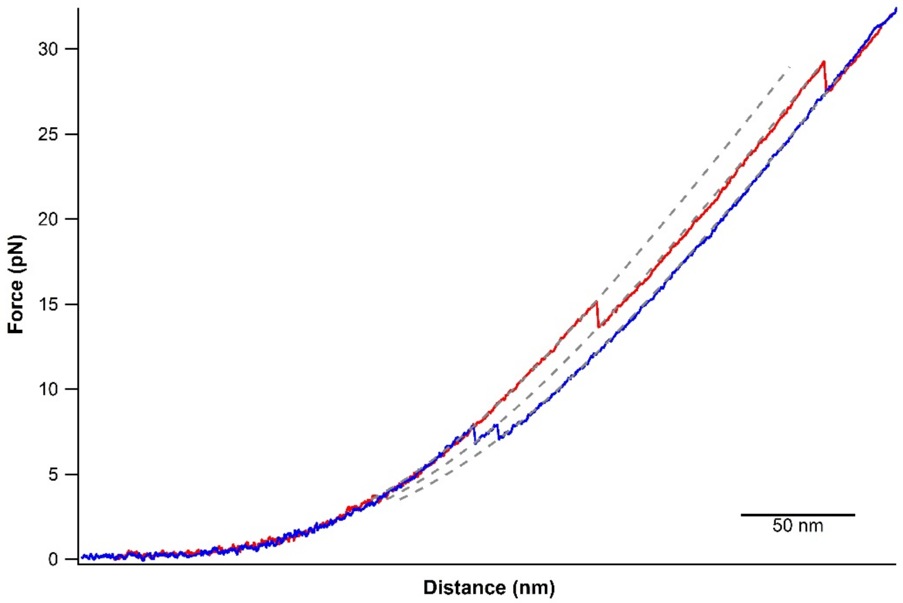

Figure S3. Mechanical unfolding and folding of two NuG2 protein domains at a pulling speed of 50 nm/s. The unfolded NuG2 domains follow the typical WLC behavior of a unfolded polypeptide chain.

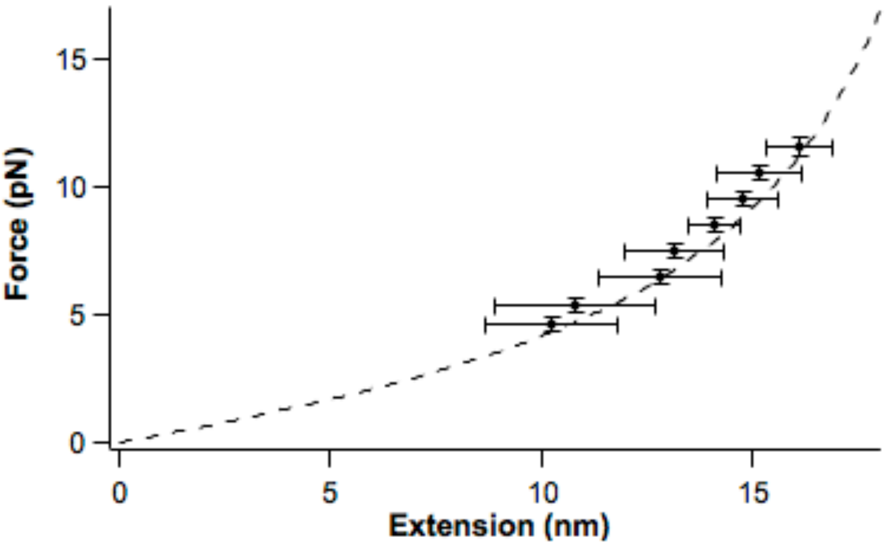

Figure S4. Force-extension relationship of TrmD Δhelix$_{45,128.}$ WLC fits yielded the persistence length of 0.8 nm and ΔLc of 25.6±0.7 nm.

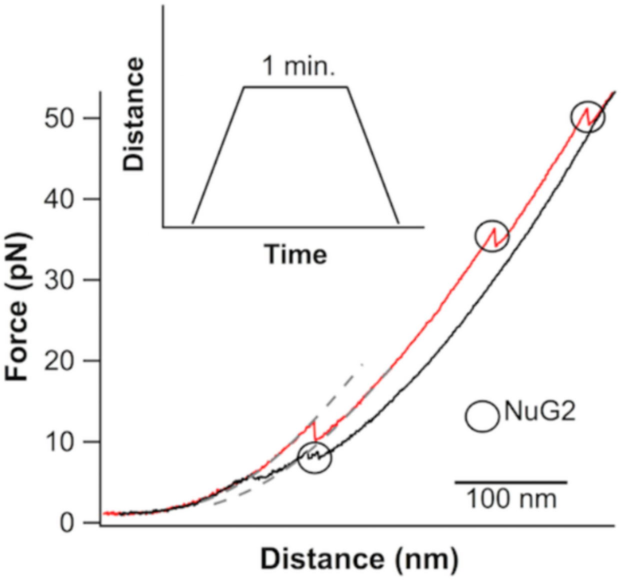

Figure S5. High tension does not inhibit the folding of $TrmD_{9,\ 246}$. After $TrmD_{9,\ 246}$ and NuG2 were unfolded (in red), the protein was stretched to a high force (~50 pN) and held at this force for 1 min. During the subsequent relaxation, $TrmD_{9,\ 246}$ was observed to fold into its native state successfully. The inset shows the experimental protocol.

Table S1. Average unfolding/folding forces and kinetic parameters for $TrmD_{9,246}$ and TrmD $\Delta helix_{45,128}$

		F_u (pN)	F_f (pN)	α_0 (s^{-1})	Δx_u (nm)	β_0 (s^{-1})	Δx_f (nm)	ΔLc (nm)
$TrmD_{9,246}$	N-U	8.0±1.8 (n=371)	4.5±0.7 (n=543)	$(3.8\pm0.5)\times10^{-3}$	2.9±0.3	$(3.3\pm0.3)\times10^{2}$	4.4±0.3	36.8±1.8
	N-I	9.5±1.3 (n=172)	N/A	$(3.6\pm0.6)\times10^{-3}$	2.9±0.4	N/D#	N/D	9.8±0.4
	I-U	8.9±1.3 (n=172)	N/A	$(3.6\pm0.3)\times10^{1}$	0.5±0.1	N/D	N/D	28.9±1.0
TrmD $\Delta helix_{45,128}$	N-U	8.2±2.3 (n=142)	4.8±0.6 (n=142)	$(2.5\pm2.2)\times10^{-1}$	0.7±0.4	$(2.5\pm0.4)\times10^{3}$	5.3±1.1	25.6±0.7

N/D: not determined

Table S2. Number of molecules, unfolding and folding events of TrmD and its mutants

	# of molecules	# of unfolding events	# of folding events
$TrmD_{9,246}$	13	543	543
TrmD $\Delta helix_{9,161}$	5	240	240
$TrmD_{45,128}$	5	76	76
TrmD $\Delta helix_{45,128}$	9	142	142

The full sequence of the engineered TrmD$_{9,246}$-(NuG2)$_2$-Cys

MRGSHHHHHHGS**MWIGIISLCPEMFRAITDYGVTGRAVKNGLLSIQSWSPR DFTHDRHRTVDDRPYGGGPGMLMMVQPLRDAIHAAKAAAGEGAKVIYL SPQGRKLDQAGVSELATNQKLILVSGRYEGIDERVIQTEIDEEWSIGDYVL SGGELPAMTLIDSVSRFIPGVLGHEASATEDSFAEGLLDSPHYTRPEVLEG MEVPPVLLSGNHAEIRRWRLKQSLGRTWLRRPELLENLALTEEQARLLA EFKTEHAQ**RS*MDTYKLVIVLNGTTFTYTTEAVDAATAEKVFKQYANDNGVDGEW TYDDATKTFTVTE*RS*MDTYKLVIVLNGTTFTYTTEAVDAATAEKVFKQYANDNGVD GEWTYDDATKTFTVTE*RSGGTKC

The sequence in bold encodes TrmD$_{9,246}$ and the sequence in italic corresponds to NuG2 domains. Cysteine residues designed as the stretching sites are underlined.
